# Circulating microRNAs in patients with hormone receptor-positive, metastatic breast cancer treated with dovitinib

**DOI:** 10.1186/s40169-017-0169-y

**Published:** 2017-10-04

**Authors:** Narayan Shivapurkar, Eveline E. Vietsch, Erin Carney, Claudine Isaacs, Anton Wellstein

**Affiliations:** Department of Oncology, Lombardi Comprehensive Cancer Center, Georgetown University, 3970 Reservoir Road NW, Washington, DC, 20057 USA

**Keywords:** MicroRNA, Liquid biopsy, Tyrosine kinase inhibitor, Breast cancer, Clinical trial

## Abstract

**Background:**

Serial analysis of biomarkers in the circulation of patients undergoing treatment (“liquid biopsies”) can provide new insights into drug effects. In particular the analysis of cell-free, circulating nucleic acids such as microRNAs (miRs) can reveal altered expression patterns indicative of mechanism of drug action, cancer growth, and tumor–stroma interactions.

**Results:**

Here we analyzed plasma miRs in patients with hormone receptor positive, metastatic breast cancer with prior disease progression during aromatase inhibitor therapy (n = 8) in a phase I/II trial with the multiple tyrosine kinase inhibitor dovitinib (TKI258). Plasma miR levels were measured by quantitative RT-qPCR before and after treatment with dovitinib. A candidate miR signature of drug response was established from a 379 miR screen for detectable plasma miRs as well as from the published literature. Changes in miR expression patterns and tumor sizes were compared. In this analysis we identified miR-21-5p, miR-100-5p, miR-125b-5p, miR-126-3p, miR-375 and miR-424-5p as potential indicators of a response to dovitinib. The altered expression patterns observed for the six circulating miRs separated patients with resistant disease from those with drug responsive disease. There was no relationship between adverse effects of dovitinib treatment and identifiable changes in miR patterns.

**Conclusion:**

We conclude that changes in the expression patterns of circulating miRs can be indicators of drug responses that merit prospective studies for validation.

**Electronic supplementary material:**

The online version of this article (doi:10.1186/s40169-017-0169-y) contains supplementary material, which is available to authorized users.

## Background

One of the hallmarks of cancer is the oncogenic activation of receptor tyrosine kinases (RTKs) that control cell growth and survival [1–3]. In addition to the autocrine cancer cell-autonomous effects, RTKs mediate the paracrine crosstalk between tumor cells and host stroma that controls fibrosis, tumor angiogenesis and the immune environment [4–8]. Thus, small molecule kinase inhibitors or antibodies that target ligands or receptors have become a mainstay of RTK targeted cancer therapy.

A recently added kinase inhibitor is dovitinib (TKI258 or CHIR-258), an orally available inhibitor of multiple RTKs that include FGFR1, FGFR3, VEGFR, KIT, and PDGFRβ with IC_50_ values < 30 nmol/L [9]. Integrated analysis of clinical and preclinical studies indicates that inhibition of FGFR signaling by dovitinib disrupts the paracrine interaction between prostate cancer and stromal cells and thus mediates the antitumor effect [10]. In some men with metastatic prostate cancer, dovitinib treatment led to improvements in bone scans and lymphadenopathy [10]. Furthermore, in pretreated patients with metastatic renal cell carcinoma, dovitinib showed significant antitumor activity in a phase-I study [11]. Moreover, in patients with metastatic renal cell carcinoma that were previously treated with a VEGFR tyrosine kinase inhibitor and an mTOR inhibitor, dovitinib treatment resulted in two partial responses (3.6%) and 29 stable diseases (52.7%) in a phase-II setting [12]. In a phase I/II and pharmacodynamic study in patients with advanced melanoma dovitinib was found to decrease levels of soluble VEGFR2 in plasma, consistent with FGFR and VEGFR inhibition [13]. Additionally, melanoma patients showed dose dependent changes in the vascularity of liver metastases after 2 days of dovitinib treatment [13]. Finally, in patients with breast cancer dovitinib showed more antitumor activity in tumors with high levels of *FGFR1* amplification. In the *FGFR1*-amplified breast cancer group, a 20.2% reduction in tumor size was found after dovitinib but no reduction in tumors with less than six copies of *FGFR1* [14].

Blood based molecular analyses (“liquid biopsies”) are used for serial monitoring of cancer progression as well as the response to treatment [15]. Here we focus on the analysis of microRNAs (miRs) in the circulation, which are transcribed, processed, packaged and released from cells in normal and in diseased tissues as part of the local and at-a-distance cellular crosstalk [16, 17]. Distinct alterations in circulating miRs can reflect dysregulation of cell proliferation, immunity and stromal interactions. As shown in numerous studies, circulating miRs can serve as predictors of cancer outcome [18–22] and may allow for a real-time assessment of treatment responses after surgical resection [23], chemotherapy [24–27] or pathway targeted therapy [28]. Drug treatment impacts both cancerous lesions and the host tissues. Therefore, changing patterns of miRs in the circulation should reflect the impact of the treatment on the cancer lesion as well as the host organism and makes circulating miRs suitable biomarkers [15].

Here we analyzed cell-free miRs in the plasma of breast cancer patients in a phase I/II trial of dovitinib. We hypothesized that changes in circulating miR patterns can indicate dovitinib treatment responses, resistance to treatment, or adverse effects.

## Methods

### Patient eligibility

Post-menopausal women with hormone receptor positive, HER2 negative metastatic breast cancer 18 years or older were included in this phase I/II open-label single arm trial evaluated dovitinib in combination with an aromatase inhibitor (AI), i.e. anastrozole, exemestane or letrozole. Index tumors had to be 10 mm or greater on a CT scan and had previously been sensitive to endocrine therapy followed by disease progression after > 2 years of adjuvant endocrine therapy. At entry into the study evidence of disease resistance to an AI, defined as documented disease progression while receiving an AI, or development of disease recurrence < 6 months after completing adjuvant therapy with an AI. Patients had to have a good performance status (eastern Cooperative Group 0–1), adequate organ function and had to have life expectancy of > 3 months. All patients gave written consent for the clinical trial protocol that had been approved by the IRB at Medstar Georgetown University Hospital (IRB #2010-535). The trial is registered at clinicaltrials.gov as NCT01484041.

### Study design and treatment

Dovitinib was given at an oral dose of 500 mg daily 5 days on/2 days off in combination with the standard dose of AI (either anastrozole 1 mg daily, exemestane 25 mg daily, or letrozole 2.5 mg daily). One treatment cycle was defined as 4 weeks. After each cycle, peripheral venous blood samples were collected and after removal of personal identifiers plasma was separated and stored at – 80 °C until further processing. For the phase I portion of the study, if more than 2 dose-limiting-toxicities (DLT) were seen in the first 6 subjects, the dose of dovitinib was reduced to 400 mg daily 5 days on/2 days off in combination with the fixed dose of AI. Eventually 3 subjects remained on the initial dose of 500 mg and dose de-escalation was performed for 9 patients that were treated at 400 mg after the first cycle. Tumor responses to treatment with dovitinib were evaluated every 2 cycles (8 weeks) as determined by CT scans of patients’ index lesions. The trial was stopped after 12 patients had enrolled when the decision was made to discontinue development of dovitinib. Plasma samples before and after treatment were collected for only 8 out of 12 patients due to early exit from the study by 2 patients, and logistical difficulties.

### Circulating miR analysis

Plasma miR extraction and RT-qPCR analysis was conducted as previously reported [22, 29]. Briefly, two replicates of 170 µL of plasma were thawed, mixed with 5 volumes of Qiazol lysis reagent and vortexed. For one patient, 500 µL plasma pre and post treatment was used to extract miRs for a broader analysis of 379 miRs in an array format. The miRs were extracted with chloroform and the aqueous phase was further processed using the miRNeasy Mini kit (Qiagen, Valencia, CA). MiRs were converted to cDNA using the miScript II RT kit (Qiagen Valencia, CA, cat. # 218160). This kit contains a miScript HiSpec buffer MiR to enable either mature miRNA profiling (using miScript miRNA PCR Arrays) or mature miRNA quantification using individual miScript Primer Assays. In this reverse transcription reaction with miScript HiSpec Buffer, mature miRNAs are polyadenylated by poly(A) polymerase and converted into cDNA by reverse transcriptase with oligo-dT priming. The cDNA is then used for real-time PCR quantification of mature miRNA expression. MiR expression was quantified by qRT-PCR, using the SYBR green PCR Master mixture (Qiagen cat. # 218073) in an ABI7900HT Real-Time PCR system using (Applied Biosystems, Foster City, CA) with 95 °C for 15 min followed by 40 cycles (94 °C for 15 s, 55 °C for 30 s and 72 °C for 30 s) followed by a melting curve step to evaluate specificity of amplification. A broad miR expression array (379 miRs + U6) was performed for global miR profiling using one randomly selected patient (# 101) that required 500 µL plasma at baseline and post dovitinib treatment (Qiagen Valencia, CA). The miR expression values from the array were normalized to the mean expression level of all miRs in the respective sample, to adjust for the different quality of RNA preservation and extraction. MiR specific primers were used for the panel of six miRs selected for further study (Qiagen Valencia, CA). Expression values were calculated using the comparative C*t* method. Levels of a panel of six selected signature of miRs were measured in the plasma of eight patients before and after treatment and normalized to U6 levels [30, 31]. The data was processed with Prism 5.0 Graphpad software for t test analysis and the display of data. Reproducibility of the miRNA assays within and across studies supports that these assays are specific for mature miRNAs, as also claimed by the manufacturer.

### Statistical analysis

Principal component analysis (PCA) and the agglomerative hierarchical clustering were performed with the miR measurements from all 8 patients and included the levels of the selected six miRs before and after treatment. The XLSTAT Version 2014.6.01 from Addinsoft was used for these analyses. From the PCA and clustering of the miR measurements, distinct patterns of patients’ responses to the drug treatment were visualized and patients were assigned to distinct response groups. The investigators performing the miR panel selection, and the PCA-based assignment of patients to treatment response groups were blinded to the clinical outcomes and the measurements of tumor sizes during the trial. Expression level changes of the six selected miRs between groups of patients based on their response to dovitinib were compared to baseline levels in Prism Graphpad Version 5.03 by using unpaired Student *t*-test.

## Results

### Patients undergoing dovitinib treatment

Out of the 12 patients enrolled in the trial, 6 patients came off study for disease progression, 4 patients for toxicity, and 2 patients chose to discontinue treatment. Patient enrollment stopped after the 12th patient due to limited clinical benefits and the company’s strategic decision not to develop the drug further. Sufficient amounts and quality of pre- and post-treatment plasma samples for miR analysis were available from 8 of 12 patients. The comparative miR analysis was done with the pre-treatment and the earliest available plasma sample after initiation of dovitinib treatment. For 2 out of 8 patients blood samples were not collected after the first cycle of dovitinib (patients 101, 301), therefore miRs were analyzed after the second cycle of dovitinib for these patients.

**Fig. 1:**
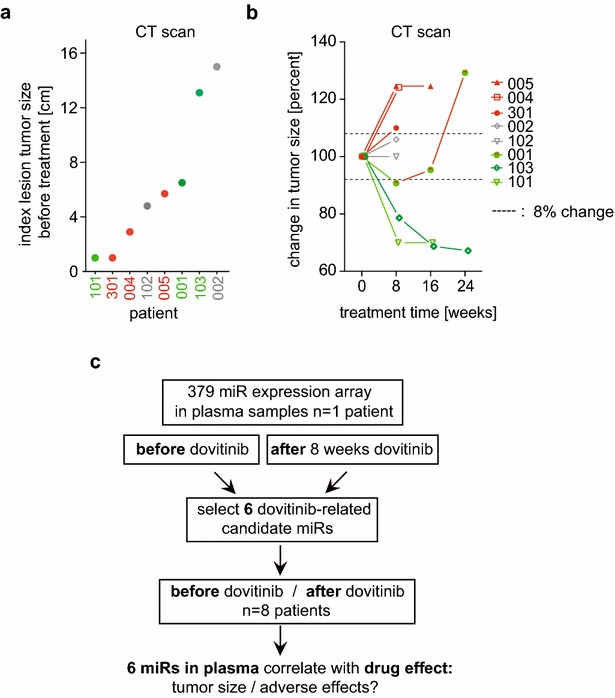
Breast cancer lesion sizes, treatment responses and design of the miR analysis. **a** Patient numbers are color coded to indicate changes in tumor sizes after 8 weeks of treatment (red = increase, green = decrease, grey = no change). The sizes of the index lesions were measured by CT before treatment with dovitinib (baseline). **b** Change in tumor size of the index lesion during the course of dovitinib treatment. **c** Flow chart of plasma miR analysis design. From a genome wide miR expression array with samples collected before and after dovitinib treatment in a single patient, 6 signature miRs were selected and tested for their indication of a dovitinib response in 8 patients. Further criteria for selection of the miRs are provided in the “Results” section

The clinical characteristics of the 8 patients studied as well as their treatments and adverse effects are compiled in Table 1. Baseline index tumor size of target lesions before dovitinib treatment, obtained from the computerized tomography (CT) measurements are shown in Fig. 1a. The change in tumor sizes over the course of dovitinib treatment is shown in Fig. 1b. Tumor responses after 8 weeks of treatment were not correlated with tumor sizes at baseline. The change in index tumor size based on the CT measurements after 8 weeks of treatment were used to separate patients into subgroups, i.e. tumors that had increased in size by more than 8% (n = 3; red symbols), decreased in size by more than 8% (n = 3; green symbols), and those that were not changed (n = 2; grey symbols) relative to baseline.

**Table 1:** Patients with hormone positive metastatic breast cancer treated with dovitinib

Pt #	Age	Index lesion tumor size at baseline (cm)	Aromatase inhibitor	Total # cycles of dovitinib	Dovitinib dose (mg)	AE after first cycle	Change in tumor size after 8 weeks dovitinib (%)	Sites of metastases
001	61	6.5	Exemestane	6	500	Fatigue GI: nausea, dry mouth Neuromusc: myalgia, dizziness	− 9.23	Lymph nodes, bone, skin/soft tissue
002	32	15	Anastrozole	1	500	Fatigue GI: nausea, vomiting, diarrhea, dehydration, poor appetite Neuromusc: headaches, blurred vision, lightheadedness Liver: ALP↑	+ 6.0	Lymph nodes, lung, bone
004	65	2.9	Letrozole	2	400	Fatigue, hypertension, bone pain GI: anorexia, nausea, dyspepsia	+ 24.14	Lung, liver
005	58	5.7	Exemestane	3	400	Fatigue GI: nausea, vomiting, anorexia, dysgeusia Neuromusc: muscle weakness Liver: ALP↑, AST↑	+ 24.56	Lymph nodes, soft tissue, liver
101	64	1	Anastrozole	4	500	Serum amylase↑, creatine↑, magnesium↑ Liver: GGT↑	− 30.0	Lymph nodes, bone
102	57	4.8	Letrozole	2	400	GI: nausea Liver: GGT↑	0	Lymph nodes, bone
103	52	13.1	Anastrozole	7	400	GI: diarrhea Neuromusc: back pain Liver: GGT↑, ALT↑, ALP↑	− 21.37	Lymph nodes, bone, pleura, liver
301	51	1	Letrozole	3	400	Hypokalemia GI: diarrhea, dyspepsia Neuromusc: arthralgia Liver: triglycerides↑, ALT↑, ALP↑, GGT↑ Anemia, Cholesterol↑	+ 10	Lung

*pt* patient, *cm* centimeter, *mg* milligram, *AE* adverse events, *GI* gastro intestinal, *Neuromusc*, neuro muscular, *PD* progressive disease, *ALP* alkaline phosphatase, *ALT* alanine aminotransferase, *GGT* gamma-glutamyl transpeptidase, *AST* aspartate aminotransferase, *w* weeks

### Selection of circulating microRNAs impacted by dovitinib treatment

**Fig. 2:**
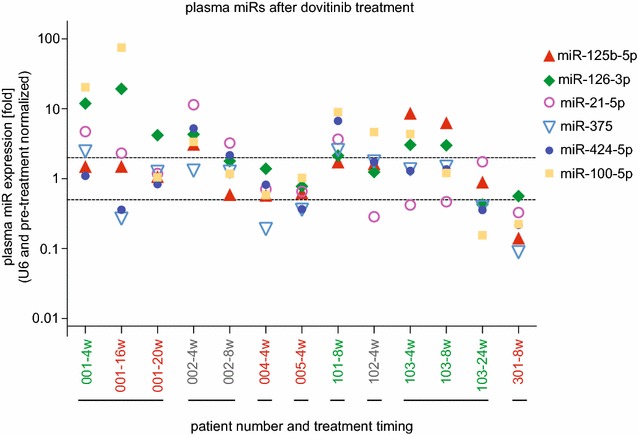
Effect of dovitinib treatment on plasma miR levels of patients with metastatic breast cancer. MiRs were measured in plasma samples of patients collected before and after dovitinib treatment. The change in expression levels of six miRs relative to U6 in 8 patients at different treatment times (in weeks; w) is shown. The dashed lines indicate twofold up- or down-regulation of miR expression levels after treatment. The numbers on the x-axis indicate the patients. The color code of patients indicates changes in tumor sizes during treatment (red = increase, green = decrease, grey = no change)

We followed the approach and rationale of our recent study on the selection of a panel of circulating miRs in patient samples [22]: Six miRs were selected based on the following criteria in order of priority: (a) abundance of the miR: Measurable levels in the plasma before and after treatment; (b) references in the published literature that relate the respective miR to breast cancer and/or to FGF, VEGF or PDGF signaling, i.e. the pathways targeted by dovitinib; (c) detectable change relative to dovitinib treatment by at least threefold up or down. The workflow of the miR analysis is shown in Fig. 1c. To support the selection of a small panel of miRs, a screen for 379 miRs and U6 was run on paired plasma samples that were obtained before and after treatment of a patient that was randomly selected and also contained sufficient amounts of U6 for the assay. In this qRT-PCR based miR expression array, 135 of 379 miRs were detectable above the threshold expression level in samples collected before and after dovitinib treatment (Additional file 1: Table S1). After data normalization using the mean expression value of all miRs detectable in the respective samples, 60 miRs showed a down-regulation and 33 miRs an upregulation of at least threefold after the treatment. Six circulating miRs i.e. miR-21-5p, miR-100-5p, miR-125b-5p, miR-126-3p, miR-375 and miR-424-5p were selected based on the three criteria that were outlined above. The expression of the 6 miRs was then measured by qRT-PCR before and after dovitinib in all patients. The change in expression after treatment relative to baseline is shown in Fig. 2. A > 100-fold concentration range of these six miRs was found in the circulation of the patients. The response to dovitinib was distinct amongst patients indicating differential drug effects between patients.

### Plasma miR levels as indicators of response to dovitinib treatment

**Fig. 3:**
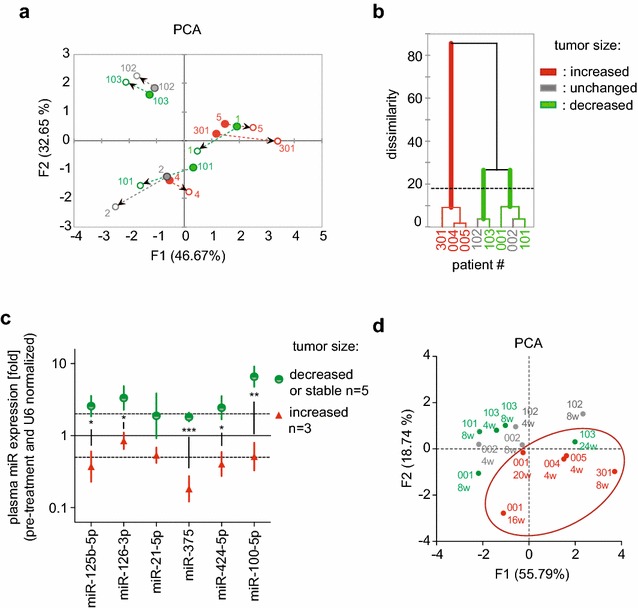
Expression patterns of circulating miRs separate patients based on the response to dovitinib treatment. **a** Principal component analysis of the expression of six miRs before treatment (closed circles) and after treatment (open circles): patients 001, 002, 004, 005, 102, 103: 4 weeks; patients 101, 301: 8 weeks. The arrows indicate the before-to-after direction of the change. Patients that are separated by color code to indicate changes in tumor sizes after 8 weeks treatment (red = increase, green = decrease, grey = no change). **b** Cluster analysis of the miR expression changes after 4 or 8 weeks treatment with dovitinib. The dashed line indicates significant differences (Euclidian distance) between 3 patient groups. **c** Changes in six circulating miRs early after dovitinib treatment (4 weeks, except patient 101 and 103 at 8 weeks) based on tumor responses. The fold change in expression after treatment relative to U6 is indicated. Error bars = SEM, *p < 0.05; **p = 0.0032; ***p = 0.0006 by unpaired t-test. **d** Principal Component Analysis of the changes in expression of six miRs after dovitinib at multiple time points. The red circle includes patients with dovitinib resistant tumors. Patient 103 at 24 weeks is the exception in this group

To evaluate whether the circulating miRs could indicate patient’s responses based on the change in expression after dovitinib, we used PCA and clustering analysis. The PCA in Fig. 3a indicates the pattern changes per patient changes based on the miR levels before and after the initial dovitinib treatment (4–8 weeks). The closed circles in the PCA (Fig. 3a) indicate the expression signature of six miRs at baseline per patient. We found three categories of patients based on the change in miR levels after the initial dovitinib treatment as indicated by the direction of the arrows pointing from baseline to post treatment. A cluster analysis of miR patterns (Fig. 3b) separated the patients into two major groups with one group split further into two subgroups (p < 0.05). PCA and cluster analyses of miR expression patterns were carried out by investigators that were blinded to the disease outcome. After completion of these analyses, the patient treatment responses were matched to the miR response data and are shown with a color code indicative of treatment responses (see Fig. 1b). The three patients with tumors that were resistant to dovitinib treatment showed distinct changes of plasma miR expression in the PCA and in the cluster analysis relative to patients with stable disease or tumor regression in the first 8 weeks. Interestingly, the baseline miR signatures did not separate responding from non-responding patients (closed circles in Fig. 3a). Moreover, no relationship was found between the adverse effects of dovitinib listed in Table 1 and the changes in miR patterns after dovitinib.

A comparison of the changes in miR expression after initial treatment in patients with tumor progression (n = 3) versus patients with stable disease or tumor regression (n = 5) shows that 5 out of 6 miRs were differentially expressed. In patients with treatment-resistant tumors relative to patients with stable disease or sensitive tumors miR-125b-5p, -126-3p, -375, 424-5p, and -100-5p were significantly down-regulated after the initial dovitinib treatment (Fig. 3c). This suggests that miR patterns after initial treatment can serve as response indicators. This could then support a decision to continue or terminate a planned treatment. For example, patient 001 showed a 9.23% reduction in tumor size after 2 cycles of dovitinib. However, acquired resistance became evident on the CT measurements at 24 weeks (Fig. 1b). The lack of a response based on CT measurements is obvious at 24 weeks but was already evident based on the circulating miR patterns at 16 weeks, as seen in the PCA (Fig. 3d). Additionally, patient 103 had a long-term anti-tumor response to dovitinib (Fig. 1b). However, when this anti-tumor response began to diminish at 24 weeks, the circulating miR pattern was already similar to that of the resistant group of patients (Fig. 3d). Thus, the change in expression levels of six miRs selected here may serve as biomarkers of anti-tumor responses during treatment with a TKI in patients with metastatic breast cancer.

## Discussion

Here we report that distinctly altered patterns of circulating miR expression are observed in patients after treatment with dovitinib. Plasma miRs have a major potential as cancer biomarkers [32–34]. MiRs are deregulated as a result of the uncontrolled cell proliferation, stromal remodeling and immune regulation that define cancer and are stably exported into the circulation. Distinct alteration in circulating miRs reflects dysregulation of cell growth and stroma recruitment and the impact of therapy. Generally speaking, the six miRs selected as potentially informative miRs for a dovitinib effect will reflect the effects of the drug on the host as well as on the tumor and serial blood sampling relative to treatments may capture the dynamics of these events.

The function of the five miRs that show a significant change in dovitinib-responsive tumors (Fig. 3c) and a possible relation with drug efficacy is discussed next. Recent work indicates that elevated miR-125b expression predicts poor prognosis in breast cancer and is a candidate therapeutic target in AI-resistant breast cancers [35]. Interestingly, miR-125b expression is transiently induced in endothelial cells upon stimulation with VEGF or by ischemia [36]. Also, miR-125b inhibits translation of vascular endothelial (VE)-cadherin mRNA, in vitro tube formation by endothelial cells, and induced nonfunctional blood vessel formation in vivo resulting in inhibition of xenograft tumor growth [36]. This matches with an antiangiogenic effect of dovitinib treatment expected from an inhibitor that targets FGF and VEGF pathways.

MiR-126 is considered the prototype of an endothelial-specific miRNA. VEGF-A is a target for miR-126 and studies suggested that miR-126 could suppress tumor growth and tumor angiogenesis through VEGF-A signaling [37, 38]. Others described miR-126 as an independent suppressor of the sequential recruitment of mesenchymal stem cells and inflammatory monocytes into breast cancer stroma to inhibit lung metastasis [39]. This study also demonstrated a correlation between miR-126 downregulation and poor metastasis-free survival of breast cancer patients [39]. The expression of miR-126 has been shown to be downregulated in breast metastases [40], as well as different cancers acting as a tumor suppressor by inhibiting tumor growth [41, 42]. The treatment related upregulation in patients with dovitinib-responsive tumors fits with this role of miR-126.

Circulating miR-375 is negatively correlated with disease relapse and resistance to neoadjuvant chemotherapy in stage II–III breast cancer patients [43]. Wu et al. also identified miR-375 as the most significantly different miRNA, whose prevalence in the circulation appeared to reflect better clinical outcome of breast cancer [43]. miR-424 was reported to directly control the expression of FGFR1 and MAP2K1 in the human trophoblast through a discrete 3′UTR site [44]. Thus, the increase observed in patients with dovitinib-responsive tumors suggests a relationship between the altered circulating miR-424 levels and the efficacy of dovitinib towards one of its known targets, the FGF receptor pathway.

MiR-100 was the most differentially upregulated miR in patients with dovitinib-responsive tumors (Fig. 3c). It has been shown that miR-100 inhibits the maintenance and expansion of breast cancer stem cells in basal-like cancer and plays a role in cancer free-survival, as confirmed by a cohort analysis of patient tumors implicating low expression of miR-100 as a negative prognostic factor [45]. Thus, the upregulation in patients with treatment responsive tumors matches with the role ascribed to this miR.

Given the complex interplay of cancer and stroma that includes distinct drivers in different cancers as well as genetic and environmental differences in the patient, analysis of patterns of miRs can provide a more reliable read-out than individual miRs and will compensate for this heterogeneity. Also, rather than evaluating absolute levels of single miRs in patients with different genetic backgrounds, co-morbidities and lifestyle, our study suggests that it is more informative to evaluate changes in the expression patterns of a set of miRs due to therapy and during the course of the disease. Here, we assessed miR expression pattern changes in response to therapy, and evaluated whether this differs amongst patients with different courses of their disease.

As a caveat, the small size of the current study limits the potential for general conclusions. However, we have attempted to follow the guidelines of McShane et al. [46] and were surprised by the robustness of the relationship between the change in tumor size and circulating miR pattern changes. Rather than trying to find a correlation between miR pattern changes and cancer lesion treatment responses we opted to use the miR pattern changes to assign patients to different response groups in an analysis of miRs that was blinded to the patient outcome. This blinded assignment of patients to distinct response groups also strengthens the conclusions one can draw from the findings.

In conclusion, altered patterns of serially analyzed circulating miR expression patterns can indicate the impact of treatment on the whole organism as well as cancer lesion. Changes in the pattern changes after treatment will be informative on the potential long-term benefit of the therapy and thus can provide an early decision point for continuation of a given treatment [15]. The current study suggests that the use of circulating miRs for treatment monitoring could be useful in treatment decisions though a prospective trial will be necessary to unanimously confirm the utility of the approach described here.

## Additional file

**Additional file 1: Table S1.** Genome wide miR expression screen before and after dovitinib treatment. An RT-qPCR assay was performed on a paired set of one patient’s (#101) plasma samples. Abundant miRs with cycle numbers (Ct) below 32 are listed.

## Abbreviations

miR: microRNA
RTK: receptor tyrosine kinase
TKI258 or CHIR-258: dovitinib
VEGFR: vascular endothelial growth factor receptor
KIT: KIT proto-oncogene receptor tyrosine kinase
FGFR: fibroblast growth factor receptor
PDGFRβ: platelet derived growth factor receptor beta
IC_50_: half maximal inhibitory concentration
TKI: tyrosine kinase inhibitor
mTOR: mechanistic target of rapamycin
AI: aromatase inhibitor
qRT-PCR: quantitative reverse transcription polymerase chain reaction
DLT: dose-limiting-toxicities
HER2: human epidermal growth factor receptor 2
CT: computerized tomography
PCA: principal component analysis
