# Supplementary material for: Circulating microRNAs in patients with hormone receptor-positive, metastatic breast cancer treated with dovitinib
Source: Clin Transl Med. 2017 Oct 4;6:37. doi: 10.1186/s40169-017-0169-y (PMC5628092; doi:10.1186/s40169-017-0169-y)
Supplement: Supplementary file 1 — Additional file 1: Table S1. Genome wide miR expression screen before and after dovitinib treatment. An RT-qPCR assay was performed on a paired set of one patient’s (#101) plasma samples. Abundant miRs with cycle numbers (Ct) below 32 are listed. [file 40169_2017_169_MOESM1_ESM.docx]

**Supplemental Table 1. Genome wide miR expression screen before
and after dovitinib treatment.** An RT-qPCR assay was performed on a
paired set of one patient’s (#101) plasma samples. Abundant miRs with cycle numbers
(Ct) below 32 are listed.

|  | **Ct before treatment** | **Ct after treatment** |
| --- | --- | --- |
| mean Ct value | 26.08 | 28.76 |
| **miR** |  |  |
| hsa-let-7a-5p | 21.18 | 28.84 |
| hsa-let-7b-5p | 21.13 | 29.23 |
| hsa-let-7c | 24 | 27.32 |
| hsa-let-7d-3p | 24.98 | 30.29 |
| hsa-let-7d-5p | 23.88 | 28.66 |
| hsa-let-7e-5p | 23.34 | 30.57 |
| hsa-let-7f-5p | 22.39 | 28.14 |
| hsa-let-7g-5p | 23.18 | 28 |
| hsa-let-7i-5p | 24.69 | 30.52 |
| hsa-miR-1 | 26.26 | 29.68 |
| hsa-miR-100-5p | 25.84 | 25.96 |
| hsa-miR-101-3p | 28.89 | 31.75 |
| hsa-miR-105-5p | 23.97 | 28.92 |
| hsa-miR-106b-5p | 25.34 | 29.53 |
| hsa-miR-107 | 28.24 | 31.2 |
| hsa-miR-1180 | 28.44 | 30.7 |
| hsa-miR-122-5p | 24.41 | 29.22 |
| hsa-miR-124-3p | 30.76 | 29.45 |
| hsa-miR-125a-5p | 24.19 | 30.75 |
| hsa-miR-126-3p | 22.06 | 28.27 |
| hsa-miR-126-5p | 21.01 | 25.53 |
| hsa-miR-127-3p | 26.79 | 24.73 |
| hsa-miR-128 | 27.53 | 30.96 |
| hsa-miR-1290 | 27.65 | 30.44 |
| hsa-miR-130a-3p | 26.20 | 29.52 |
| hsa-miR-130b-3p | 30.16 | 28.34 |
| hsa-miR-132-3p | 28.72 | 30.42 |
| hsa-miR-132-5p | 18.40 | 22.68 |
| hsa-miR-134 | 28.10 | 25.74 |
| hsa-miR-135a-5p | 26.52 | 28.35 |
| hsa-miR-142-3p | 26.69 | 26.95 |
| hsa-miR-142-5p | 29.40 | 30.56 |
| hsa-miR-144-3p | 23.24 | 28.89 |
| hsa-miR-144-5p | 28.05 | 30.54 |
| hsa-miR-146a-5p | 23.17 | 28.52 |
| hsa-miR-146b-5p | 25.78 | 30.07 |
| hsa-miR-148a-3p | 25.57 | 28.82 |
| hsa-miR-148b-3p | 25.73 | 30.86 |
| hsa-miR-150-5p | 23.16 | 24.2 |
| hsa-miR-151a-3p | 24.99 | 30.77 |
| hsa-miR-151a-5p | 26.59 | 30.68 |
| hsa-miR-152 | 27.94 | 31.8 |
| hsa-miR-155-5p | 27.62 | 30.84 |
| hsa-miR-15b-3p | 26.59 | 30.58 |
| hsa-miR-15b-5p | 30 | 28.89 |
| hsa-miR-16-5p | 20.35 | 25.82 |
| hsa-miR-17-5p | 25.37 | 30.13 |
| hsa-miR-181a-5p | 30.10 | 28.25 |
| hsa-miR-181c-3p | 27.52 | 26.2 |
| hsa-miR-183-3p | 29.46 | 24.87 |
| hsa-miR-185-5p | 24.96 | 29.12 |
| hsa-miR-186-5p | 23.05 | 26.68 |
| hsa-miR-187-5p | 29.66 | 28.44 |
| hsa-miR-18a-3p | 27.53 | 30.61 |
| hsa-miR-18a-5p | 25.86 | 29.3 |
| hsa-miR-18b-5p | 26.56 | 30 |
| hsa-miR-191-5p | 22.90 | 26.49 |
| hsa-miR-192-5p | 28.10 | 30.63 |
| hsa-miR-193a-5p | 28.23 | 31.38 |
| hsa-miR-194-5p | 28.82 | 31.82 |
| hsa-miR-195-5p | 20.69 | 25.43 |
| hsa-miR-197-3p | 24.89 | 30.34 |
| hsa-miR-199a-3p | 23.43 | 29.72 |
| hsa-miR-19a-3p | 24.67 | 30.01 |
| hsa-miR-19b-3p | 24.57 | 28.62 |
| hsa-miR-200b-3p | 29.35 | 28.78 |
| hsa-miR-200c-3p | 29.95 | 29.95 |
| hsa-miR-200c-5p | 28.11 | 28.87 |
| hsa-miR-205-3p | 31.50 | 28.2 |
| hsa-miR-20a-5p | 24.31 | 30.28 |
| hsa-miR-20b-5p | 25.46 | 31.56 |
| hsa-miR-21-5p | 20.53 | 22.91 |
| hsa-miR-210 | 31.13 | 30.21 |
| hsa-miR-22-3p | 23.56 | 18 |
| hsa-miR-22-5p | 28.9 | 30.72 |
| hsa-miR-221-3p | 20.89 | 25.28 |
| hsa-miR-221-5p | 31.13 | 31.18 |
| hsa-miR-222-3p | 26.92 | 30.08 |
| hsa-miR-223-3p | 20.93 | 25.44 |
| hsa-miR-223-5p | 27.47 | 30.39 |
| hsa-miR-23b-3p | 24.68 | 30.7 |
| hsa-miR-24-3p | 22.73 | 28.22 |
| hsa-miR-25-3p | 22.19 | 25.1 |
| hsa-miR-25-5p | 28.73 | 30.21 |
| hsa-miR-26a-5p | 22.54 | 28.48 |
| hsa-miR-26b-5p | 23.99 | 30.02 |
| hsa-miR-27a-3p | 24.28 | 30.39 |
| hsa-miR-27b-3p | 23.96 | 31.57 |
| hsa-miR-28-3p | 28.58 | 28.57 |
| hsa-miR-29a-3p | 27.09 | 28.32 |
| hsa-miR-30a-5p | 25.49 | 30.78 |
| hsa-miR-30c-5p | 24.43 | 30.2 |
| hsa-miR-30d-5p | 24.70 | 29.46 |
| hsa-miR-30e-3p | 26.54 | 31.72 |
| hsa-miR-30e-5p | 25.31 | 28.94 |
| hsa-miR-31-5p | 31.75 | 30.52 |
| hsa-miR-320a | 22.70 | 27.08 |
| hsa-miR-320b | 26.99 | 28.49 |
| hsa-miR-324-5p | 28.80 | 30.8 |
| hsa-miR-328 | 27.10 | 25.24 |
| hsa-miR-330-3p | 30.11 | 28.12 |
| hsa-miR-342-3p | 24.57 | 26 |
| hsa-miR-34c-3p | 30.18 | 31.46 |
| hsa-miR-34c-5p | 30.76 | 32 |
| hsa-miR-361-5p | 26.24 | 31.96 |
| hsa-miR-370 | 28.46 | 27.48 |
| hsa-miR-373-5p | 28.32 | 31.92 |
| hsa-miR-374b-5p | 24.95 | 30.92 |
| hsa-miR-375 | 28.81 | 28.46 |
| hsa-miR-382-5p | 26.41 | 31.61 |
| hsa-miR-411-5p | 30.34 | 24.96 |
| hsa-miR-421 | 28.64 | 26.86 |
| hsa-miR-423-3p | 27.85 | 31.73 |
| hsa-miR-424-5p | 27.03 | 27 |
| hsa-miR-431-5p | 28.45 | 25.94 |
| hsa-miR-433 | 27.18 | 23.85 |
| hsa-miR-451a | 20.63 | 25.1 |
| hsa-miR-484 | 24.07 | 28.61 |
| hsa-miR-486-5p | 19.30 | 24.83 |
| hsa-miR-489 | 25.67 | 20.59 |
| hsa-miR-495-3p | 26.41 | 24.73 |
| hsa-miR-497-5p | 30.11 | 30.95 |
| hsa-miR-539-5p | 29.31 | 21.08 |
| hsa-miR-551b-3p | 31.74 | 30.66 |
| hsa-miR-652-3p | 24.95 | 31.68 |
| hsa-miR-661 | 28.82 | 27.68 |
| hsa-miR-7-5p | 25.79 | 30 |
| hsa-miR-720 | 23.97 | 31.37 |
| hsa-miR-744-5p | 24.12 | 30.61 |
| hsa-miR-92a-3p | 21.04 | 27.99 |
| hsa-miR-92b-3p | 27.24 | 30.67 |
| hsa-miR-93-5p | 25.75 | 28.65 |
| hsa-miR-99b-5p | 28.62 | 31.29 |
| RNU6-2 | 27.26 | 30.76 |
